# Supplementary material for: Prevalence of Non-Affective Psychoses in Individuals with Autism Spectrum Disorders: A Systematic Review
Source: J Clin Med. 2019 Aug 24;8(9):1304. doi: 10.3390/jcm8091304 (PMC6780908; doi:10.3390/jcm8091304)
Supplement: Supplementary file 1 [file jcm-08-01304-s001.pdf]

Appendix A. Search strategy

1	exp child development disorders, pervasive/	31618
2	Developmental Disabilities/	18993
3	pervasive development$ disorder$.tw.	1965
4	(pervasive adj3 child$).tw.	457
5	(PDD or PDDs or ASD or ASDs).tw.	23718
6	autis$.tw.	42462
7	asperger$.tw.	1999
8	kanner$.tw.	208
9	1 or 2 or 3 or 4 or 5 or 6 or 7 or 8	72990
10	exp Schizophrenia/	100958
11	exp Paranoid Disorders/	4029
12	schizophrenic.mp.	49893
13	10 or 11 or 12	112467
14	9 and 13	1809

Appendix B. Quality assessment of observational included studies through Newcastle - Ottawa Quality Assessment Scale

Cross-sectional studies [1]							
	Selection				Comparability	Outcome	
Study	Representativeness of the sample	Sample size	Non-respondents	Ascertainment of the exposure (risk factor) (maximum 2 stars)	Comparability of outcome groups (maximum 2 stars)	Assessment of the outcome (maximum 2 stars)	Statistical test
Bakken et al., 2010	*	*	-	**	-	*	-
Hofvander et al., 2009	-	-	-	**	*	*	*
Lugnegard et al., 2011	*	-	-	**	-	*	-
Stahlberg et al., 2004	*	*	-	**	*	*	-

Case-control studies / Case series studies							
	Selection				Comparability	Exposure	
Study	Case definition	Representativeness of the cases	Selection of controls	Definition of controls	Comparability of cases and controls (maximum 2 stars)	Ascertainment of exposure (maximum 2 stars)	Non-response rate
Abdallah et al., 2011	*	*	*	*	*	**	*
Guinchat et al., 2015 [2]	*	-	-	-	-	*	-
Joshi et al., 2010	*	*	*	*	*	**	-
Mouridsen et al., 2007a	*	*	*	*	**	**	*
Mouridsen et al., 2007b	*	*	*	*	**	**	*
Raja et al., 2010 [2]	*	-	-	*	-	*	-
Volkmar et al., 1991 [2]	*	*	-	-	-	*	-

Cohort studies							
	Selection				Comparability	Outcome	
Study	Representativeness of the exposed	Selection of the non-exposed	Ascertainment of exposure	Demonstration that outcome of interest	Comparability of cohorts (maximum	Assessment of oucome	Adequacy of follow up

	cohort	cohort		was not present at start of study	2 stars)		(maximum 2 stars)
Billstedt et al., 2005	-	*	*	-	-	-	**
Eaves et al., 2008	*	-	*	-	-	-	*
Hutton et al., 2008	*	-	*	-	-	*	**

[1] Quality assessment of cross-sectional included studies performed through Newcastle - Ottawa Quality Assessment Scale adapted for cross sectional studies (Modesti 2016)
Legend: NA: Not applicable for evaluating the quality in relation to the outcomes of our study
[2] Guinchat et al. (2015) and Volkmar et al. (1991) are case-series studies, without control groups; Raja et al. 2010 is a case-series study, with a control non-selected group.

References

1. Bakken TL, Helverschou SB, Eilertsen DE, Heggelund T, Myrbakk E, Martinsen H (2010) Psychiatric disorders in adolescents and adults with autism and intellectual disability: a representative study in one county in Norway. Res Dev Disabil 31(6):1669–1677
2. Hofvander B, Delorme R, Chaste P, Nydén A, Wentz E, Ståhlberg O, Gillberg C (2009) Psychiatric and psychosocial problems in adults with normal-intelligence autism spectrum disorders. BMC Psychiatry 9(1):35
3. Lugnegard T, Hallerback MU, Gillberg C (2011) Psychiatric comorbidity in young adults with a clinical diagnosis of Asperger syndrome. Res Dev Disabil 32(5):1910–1917. doi: 10.1016/j. ridd.2011.03.025
4. Stahlberg O, Soderstrom H, Rastam M, Gillberg C (2004) Bipolar disorder, schizophrenia, and other psychotic disorders in adults with childhood onset AD/HD and/or autism spectrum disorders. J Neural Transm 111(7):891–902
5. Abdallah MW, Greaves-Lord K, Grove J, Nørgaard-Pedersen B, Hougaard DM, Mortensen EL. Psychiatric comorbidities in autism spectrum disorders: findings from a Danish Historic Birth Cohort. Eur Child Adolesc Psychiatry. 2011 Dec;20(11-12):599-601. doi: 10.1007/s00787-011-0220-2. Epub 2011 Oct 5. PubMed PMID: 21971944.
6. Guinchat V, Cravero C, Diaz L, Périsse D, Xavier J, Amiet C, Gourfinkel-An I, Bodeau N, Wachtel L, Cohen D, Consoli A. Acute behavioral crises in psychiatric inpatients with autism spectrum disorder (ASD): recognition of concomitant medical or non-ASD psychiatric conditions predicts enhanced improvement. Res Dev Disabil. 2015 Mar;38:242-55. doi: 10.1016/j.ridd.2014.12.020. Epub 2015 Jan 7. PubMed PMID: 25575287.
7. Joshi G, Petty C, Wozniak J, Henin A, Fried R, Galdo M, Biederman J (2010) The heavy burden of psychiatric comorbidity in youth with autism spectrum disorders: a large comparative study of a psychiatrically referred population. J Autism Dev Disord 40(11):1361–1370
8. Mouridsen S, Rich B, Isager T (2008a) Psychiatric disorders in adults diagnosed as children with atypical autism. A case control study. J Neural Transm 115(1):135–138
9. Mouridsen SE, Rich B, Isager T (2008b) Epilepsy and other neurological diseases in the parents of children with infantile autism. A case control study. Child Psychiatry Hum Dev 39(1):1–8. doi: 10.1007/s10578-007-0062-9
10. Raja M, Azzoni A. Autistic spectrum disorders and schizophrenia in the adult psychiatric setting: diagnosis and comorbidity. Psychiatr Danub. 2010 Dec;22(4):514-21. PubMed PMID: 21169891.
11. Volkmar FR, Cohen DJ. Comorbid association of autism and schizophrenia. Am J Psychiatry. 1991 Dec;148(12):1705-7. PubMed PMID: 1957933.
12. Billstedt E, Gillberg C, Gillberg C (2005) Autism after adolescence: population-based 13-to 22-year follow-up study of 120 individuals with autism diagnosed in childhood. J Autism Dev Disord 35(3):351–360
13. Eaves LC, Ho HH (2008) Young adult outcome of autism spectrum disorders. J Autism Dev Disord 38(4):739–747

14. Hutton J, Goode S, Murphy M, Le Couteur A, Rutter M. New-onset psychiatric disorders in individuals with autism. Autism. 2008 Jul;12(4):373-90. doi: 10.1177/1362361308091650. PubMed PMID: 18579645.

Appendix C. References for included and excluded studies.

REFERENCES FOR INCLUDED STUDIES

References included through database searching:

1. Abdallah MW, Greaves-Lord K, Grove J, Nørgaard-Pedersen B, Hougaard DM, Mortensen EL. Psychiatric comorbidities in autism spectrum disorders: findings from a Danish Historic Birth Cohort. Eur Child Adolesc Psychiatry. 2011 Dec;20(11-12):599-601. doi: 10.1007/s00787-011-0220-2.
2. Guinchat V, Cravero C, Diaz L, Périsse D, Xavier J, Amiet C, Gourfinkel-An I, Bodeau N, Wachtel L, Cohen D, Consoli A. Acute behavioral crises in psychiatric inpatients with autism spectrum disorder (ASD): recognition of concomitant medical or non-ASD psychiatric conditions predicts enhanced improvement. Res Dev Disabil. 2015 Mar;38:242-55. doi: 10.1016/j.ridd.2014.12.020.
3. Hutton J, Goode S, Murphy M, Le Couteur A, Rutter M. New-onset psychiatric disorders in individuals with autism. Autism. 2008 Jul;12(4):373-90. doi: 10.1177/1362361308091650.
4. Raja M, Azzoni A. Autistic spectrum disorders and schizophrenia in the adult psychiatric setting: diagnosis and comorbidity. Psychiatr Danub. 2010 Dec;22(4):514-21.
5. Stahlberg O, Soderstrom H, Rastam M, Gillberg C (2004) Bipolar disorder, schizophrenia, and other psychotic disorders in adults with childhood onset AD/HD and/or autism spectrum disorders. J Neural Transm 111(7):891–902.
6. Volkmar FR, Cohen DJ. Comorbid association of autism and schizophrenia. Am J Psychiatry. 1991 Dec;148(12):1705-7.

References included through reference screening:

7. Bakken TL, Helverschou SB, Eilertsen DE, Heggelund T, Myrbakk E, Martinsen H (2010). Psychiatric disorders in adolescents and adults with autism and intellectual disability: a representative study in one county in Norway. Res Dev Disabil 31(6):1669–1677.
8. Billstedt E, Gillberg C, Gillberg C (2005). Autism after adolescence: population-based 13-to 22-year follow-up study of 120 individuals with autism diagnosed in childhood. J Autism Dev Disord 35(3):351–360.
9. Eaves LC, Ho HH (2008) Young adult outcome of autism spectrum disorders. J Autism Dev Disord 38(4):739–747
10. Hofvander B, Delorme R, Chaste P, Nydén A, Wentz E, Ståhlberg O, Gillberg C (2009). Psychiatric and psychosocial problems in adults with normal-intelligence autism spectrum disorders. BMC Psychiatry 9(1):35.
11. Joshi G, Petty C, Wozniak J, Henin A, Fried R, Galdo M, Biederman J (2010). The heavy burden of psychiatric comorbidity in youth with autism spectrum disorders: a large comparative study of a psychiatrically referred population. J Autism Dev Disord 40(11):1361–1370.
12. Lugnegard T, Hallerback MU, Gillberg C (2011). Psychiatric comorbidity in young adults with a clinical diagnosis of Asperger syndrome. Res Dev Disabil 32(5):1910–1917. doi: 10.1016/j. ridd.2011.03.025.
13. Mouridsen S, Rich B, Isager T (2008a). Psychiatric disorders in adults diagnosed as children with atypical autism. A case control study. J Neural Transm 115(1):135–138.
14. Mouridsen SE, Rich B, Isager T (2008b). Epilepsy and other neurological diseases in the parents of children with infantile autism. A case control study. Child Psychiatry Hum Dev 39(1):1–8. doi: 10.1007/s10578-007-0062-9.

REFERENCES FOR EXCLUDED STUDIES

Full-text articles excluded because there was no outcome of interest

1. Buizer-Voskamp JE; Laan W; Staal WG; Hennekam EA; Aukes MF; Termorshuizen F; Kahn RS; Boks MP; Ophoff RA. Paternal age and psychiatric disorders: findings from a Dutch population registry. Schizophrenia Research. 129(2-3):128-32, 2011.
2. Class QA; Abel KM; Khashan AS; Rickert ME; Dalman C; Larsson H; Hultman CM; Langstrom N; Lichtenstein P; D'Onofrio BM. Offspring psychopathology following preconception, prenatal and postnatal maternal bereavement stress. Psychological Medicine. 44(1):71-84, 2014.
3. Craig JS; Hatton C; Craig FB; Bentall RP. Persecutory beliefs, attributions and theory of mind: comparison of patients with paranoid delusions, Asperger's syndrome and healthy controls. Schizophrenia Research. 69(1):29-33, 2004 Jul 01. Dykens E; Volkmar F; Glick M. Though disorder in high-functioning autistic adults. Journal of Autism & Developmental Disorders. 21(3):291-301, 1991.
4. Lugnegard T; Hallerback MU; Gillberg C. Asperger syndrome and schizophrenia: Overlap of self-reported autistic traits usingthe Autism-spectrum Quotient (AQ). Nordic Journal of Psychiatry. 69(4):268-74, 2015.
5. Mandell DS; Lawer LJ; Branch K; Brodkin ES; Healey K; Witalec R; Johnson DN; Gur RE. Prevalence and correlates of autism in a state psychiatric hospital. Autism. 16(6):557-67, 2012.

6. Merikangas AK; Calkins ME; Bilker WB; Moore TM; Gur RC; Gur RE. Parental Age and Offspring Psychopathology in the Philadelphia Neurodevelopmental Cohort. Journal of the American Academy of Child & Adolescent Psychiatry. 56(5):391-400, 2017.
7. Rumsey JM; Andreasen NC; Rapoport JL. Archives of General Psychiatry. Thought, language, communication, and affective flattening in autistic adults. 43(8):771-7, 1986 Aug.
8. Sheitman BB; Kraus JE; Bodfish JW; Carmel H. Are the negative symptoms of schizophrenia consistent with an autistic spectrum illness?. Schizophrenia Research. 69(1):119-20, 2004 Jul 01.
9. Solomon M; Ozonoff S; Carter C; Caplan R. Formal thought disorder and the autism spectrum: relationship with symptoms, executive control, and anxiety. Journal of Autism & Developmental Disorders. 38(8):1474-84, 2008.
10. Sprong M; Becker HE; Schothorst PF; Swaab H; Ziermans TB; Dingemans PM; Linszen D; van Engeland H. Pathways to psychosis: a comparison of the pervasive developmental disorder subtype Multiple Complex Developmental Disorder and the "At Risk Mental State". Schizophr Res. 2008;99(1-3):38-47.
11. Starkova L; Zmrzlikova L. Catamnestic study of children hospitalized with the diagnosis of Kanner's infantile autism]. [Czech] Ceskoslovenska Psychiatrie. 85(4):238-45, 1989.
12. Vorstman JA; Breetvelt EJ; Thode KI; Chow EW; Bassett AS. Expression of autism spectrum and schizophrenia in patients with a 22q11.2 deletion. Schizophrenia Research. 143(1):55-9, 2013.
13. Weiser M; Reichenberg A; Werbeloff N; Kleinhaus K; Lubin G; Shmushkevitch M; Caspi A; Malaspina D; Davidson M. Advanced parental age at birth is associated with poorer social functioning in adolescent males: shedding light on a core symptom of schizophrenia and autism. Schizophrenia Bulletin. 34(6):1042-6, 2008.

Full-text articles excluded because the study population did not meet our inclusion criteria

14. Akande E; Xenitidis K; Roberston MD; Gorman JM. Autism or schizophrenia: a diagnostic dilemma in adults with intellectual disabilities. Journal of Psychiatric Practice. 10(3):190-5, 2004.
15. Chaplin E; Paschos D; O'Hara J; McCarthy J; Holt G; Bouras N; Tsakanikos E. Mental ill-health and care pathways in adults with intellectual disability across different residential typesResearch in Developmental Disabilities. 31(2):458-63, 2010.
16. Konstantareas MM; Hewitt T. Autistic disorder and schizophrenia: diagnostic overlaps. Journal of Autism & Developmental Disorders. 31(1):19-28, 2001 Feb.
17. Morgan VA; Leonard H; Bourke J; Jablensky A. Intellectual disability co-occurring with schizophrenia and other psychiatric illness: population-based study. British Journal of Psychiatry. 193(5):364-72, 2008.
18. Schmock H; Vangkilde A; Larsen KM; Fischer E; Birknow MR; Jepsen JR; Olesen C; Skovby F; Plessen KJ; Morup M; Hulme O; Baare WF; Didriksen M; Siebner HR; Werge T; Olsen L. The Danish 22q11 research initiative. BMC Psychiatry. 15:220, 2015.
19. Sorensen HJ; Mortensen EL; Schiffman J; Reinisch JM; Maeda J; Mednick SA. Early developmental milestones and risk of schizophrenia: a 45-year follow-up of the Copenhagen Perinatal Cohort. Schizophrenia Research. 118(1-3):41-7, 2010 May.
20. Turygin NC; Matson JL; Adams HL; Williams LW. Co-occurring disorder clusters in adults with mild and moderate intellectual disability in residential treatment settings. Research in Developmental Disabilities. 35(11):3156-61, 2014.

Full-text articles excluded because were not primary studies (reviews)

21. Ishii T. [Differential diagnosis between pervasive developmental disorder and schizophrenia]. Nippon Rinsho - Japanese Journal of Clinical Medicine. 65(3):497-501, 2007.
22. Seltzer MM; Abbeduto L; Krauss MW; Greenberg J; Swe A. Comparison groups in autism family research: Down syndrome, fragile X syndrome, and schizophrenia. Journal of Autism & Developmental Disorders. 34(1):41-8, 2004.
23. Starling J; Dossetor D. Pervasive developmental disorders and psychosis. Current Psychiatry Reports. 11(3):190-6, 2009.

Full-text articles excluded because were not reported extractable data

24. Maibing CF; Pedersen CB; Benros ME; Mortensen PB; Dalsgaard S; Nordentoft M. Risk of Schizophrenia Increases After All Child and Adolescent Psychiatric Disorders: A Nationwide Study. Schizophrenia Bulletin. 41(4):963-70, 2015 Jul.
